# Comparative safety analysis of bevacizumab and alkylating agent in glioblastoma management – What have we learned recently?

**DOI:** 10.3389/fphar.2025.1595642

**Published:** 2025-05-15

**Authors:** Zhizhao Qu, Jiajia Zhao, Liu Yang, Yuanwei Fu, Rui Bai, Jinchuan Li, Hongqin Wang

**Affiliations:** ^1^ Department of Neurosurgery, First Hospital of Shanxi Medical University, Taiyuan, Shanxi, China; ^2^ Graduate School, Shanxi Medical University, Taiyuan, Shanxi, China; ^3^ Department of Neurology, Second Hospital of Shanxi Medical University, Taiyuan, Shanxi, China; ^4^ Department of Neurosurgery, Second Hospital of Shanxi Medical University, Taiyuan, Shanxi, China

**Keywords:** temozolomide, bevacizumab, alkylating agent, glioblastoma, FAERS, safety profile

## Abstract

**Objective:**

Alkylating agents and bevacizumab are both first-line chemotherapeutic options for the treatment of glioblastoma; however, their mechanisms of action differ substantially. This study aimed to compare the safety profiles of these two drug classes in the treatment of glioblastoma to inform clinical decision-making.

**Methods:**

Adverse events reported between the first quarter of 2004 and the fourth quarter of 2023 were analyzed using data from the FDA Adverse Event Reporting System (FAERS) database. Disproportionality analysis was employed to assess and compare the AE signals associated with bevacizumab and alkylating agents.

**Results:**

In the context of glioblastoma treatment, 3,323 adverse reports were associated with bevacizumab, 5,283 with temozolomide, and 427 with lomustine. The most frequently reported AEs for bevacizumab were fatigue (n = 276), hypertension (n = 220), and headache (n = 199). Compared to temozolomide, bevacizumab was more strongly associated with “vascular disorders,” “renal and urinary disorders,” and “hypertension.” Notably, bevacizumab appeared to offer a potential safety advantage with respect to hematological adverse events.

**Conclusion:**

Our analysis indicates that bevacizumab exhibits a distinct safety profile compared to alkylating agents, particularly demonstrating a lower incidence of hematological adverse events. Further prospective studies are warranted to validate these findings and to elucidate the underlying mechanisms responsible for the observed adverse events.

## 1 Introduction

Glioblastoma is the most aggressive and prevalent type of primary brain tumor (Dolecek et al., 2012). For over a decade, the standard treatment protocol for glioblastoma has involved maximal surgical resection, followed by concurrent radiotherapy and temozolomide chemotherapy, and subsequent adjuvant temozolomide therapy for 6–12 months (Stupp et al., 2017).

The pathology of glioblastoma is characterized by the overexpression of vascular endothelial growth factor-A (VEGF-A) (Chinot et al., 2011; Hicklin and Ellis, 2005), prompting the development of therapies targeting the angiogenic pathway (Batchelor et al., 2014). Bevacizumab, a humanized monoclonal antibody, specifically binds to VEGF and inhibits its activity (Yang et al., 2003). In 2009, bevacizumab was approved by the U.S. Food and Drug Administration (FDA) for the treatment of recurrent glioblastoma, demonstrating an improvement in progression-free survival among patients with disease progression (Friedman et al., 2009; Kreisl et al., 2009). The introduction of bevacizumab heralded a new class of anti-cancer therapies, and its immunomodulatory properties have expanded therapeutic options for glioblastoma (Garcia et al., 2020).

Although the therapeutic efficacy of both alkylating agents and bevacizumab is well established, their associated adverse effects warrant careful consideration. Temozolomide has been linked to risks such as anemia, thrombocytopenia, hepatotoxicity, nausea, and vomiting (Zhou et al., 2024). In contrast, adverse events related to bevacizumab include hypertension, embolism, hemorrhage, neutropenia, and gastrointestinal perforation (Tang et al., 2024). These adverse effects can not only compromise patient compliance but also pose significant challenges to clinicians in balancing efficacy with safety.

The FDA Adverse Event Reporting System (FAERS) is a valuable database for detecting potential safety signals associated with pharmaceutical products (Yu et al., 2021). Both alkylating agents and bevacizumab serve as first-line treatments for glioblastoma but exert their effects through distinct mechanisms. Leveraging the FAERS database and data mining techniques, this study aims to identify and compare the safety signals associated with these two therapies, ultimately providing clinicians with critical information to support informed decision-making in the management of glioblastoma.

## 2 Method

### 2.1 Study design and data acquisition

This study was designed as a retrospective analysis. The FAERS collects hundreds of millions of adverse event reports submitted by healthcare professionals, consumers, and manufacturers worldwide. We selected the FAERS database as the data source for this study to identify and compare the adverse events associated with bevacizumab and alkylating agents in the treatment of glioblastoma. The study was approved by the Ethics Committee of the First Hospital of Shanxi Medical University.

### 2.2 Data processing

The FAERS database consists of several key components, including patient demographics (DEMO), drug information (DRUG), reported adverse events (REAC), patient outcomes (OUTC), report sources (RPSR), treatment start and end dates (THER), and drug indications (INDI).

**FIGURE 1:**
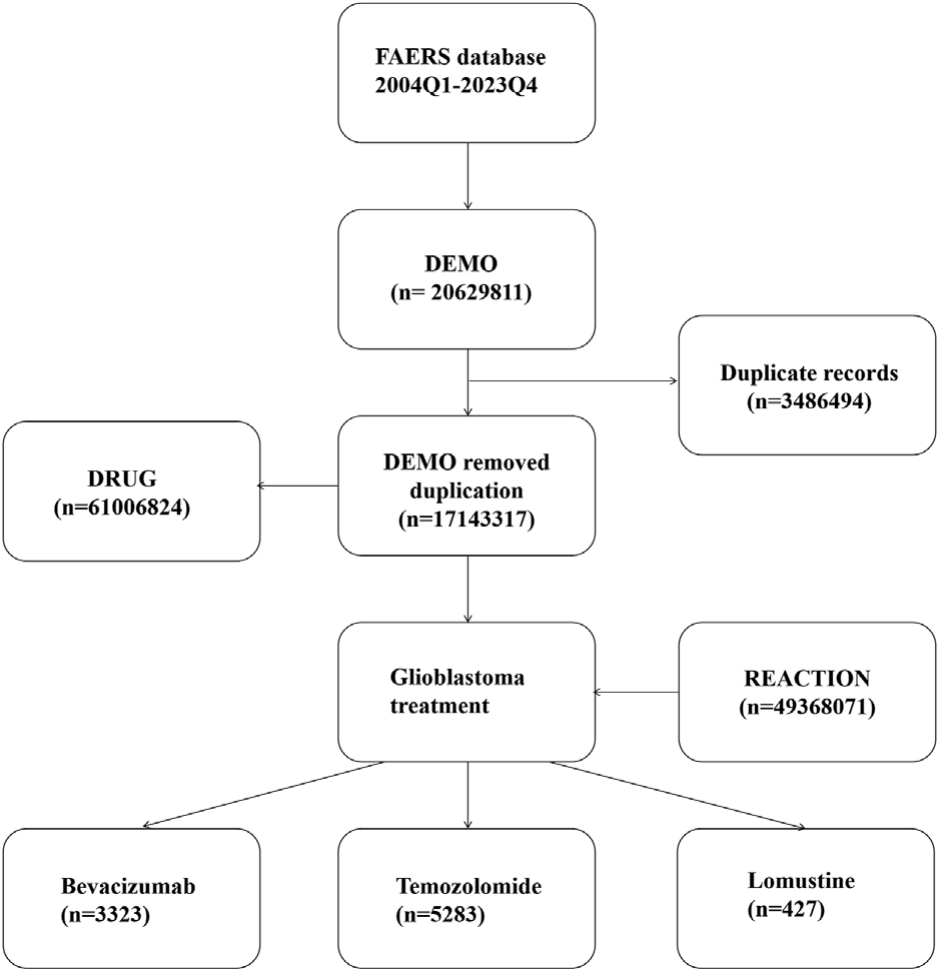
Workflow of the de-duplication process.

Since the database is updated quarterly, we implemented a de-duplication process in accordance with FDA guidelines: for identical CASEIDs, the most recent FDA_DT was retained; if both CASEID and FDA_DT were identical, the record with the larger PRIMARYID was selected. Additionally, to ensure data completeness and accuracy, we excluded entries listed in the ‘deleted cases’ files (Services USD, 2024). The de-duplication process is illustrated in Figure 1.

The study population consisted of patients whose drug indication was recorded as glioblastoma. The drugs of interest were bevacizumab, temozolomide, and lomustine. Synonyms were considered during data retrieval:

Bevacizumab: bevacizumab, anti-VEGF monoclonal antibody, bevacizumab-awwb.
Temozolomide: methazolastone, temozolodida, temozolomid, temozolomida, temozolomide, temozolomide, temozolomidum.
Lomustine: lomustina, lomustine, lomustinum; Drug identification in the DRUG table was performed through fuzzy matching of the ‘prod_ai’ and ‘drugname’ fields. To ensure that adverse events were drug-related, only reports where the “role_cod” was coded as “Primary Suspect” or “Secondary Suspect” were included.

### 2.3 Signal detection and statistical analysis

In this study, signal detection was conducted using the reporting odds ratio (ROR) method. A 2 × 2 contingency table was constructed based on the number of reported adverse events for the target drug and other drugs. The ROR and its 95% confidence interval (CI) were calculated as follows:

$$\text{ROR}=\frac{a/c}{b/d}$$

$$95\%\text{CI}=e^{In\left(ROR\right)\pm 1.96\sqrt{\frac{1}{a}+\frac{1}{b}+\frac{1}{c}+\frac{1}{d}}}$$

where:

a = number of reports of the target event with the target drug;

b = number of reports of other events with the target drug;

c = number of reports of the target event with other drugs;

d = number of reports of other events with other drugs.

Although the ROR method remains applicable when comparing a target drug to a control drug, a positive ROR result in this context should not be interpreted as a confirmed safety signal. Instead, it reflects the relative risk of specific adverse events between drugs at different classification levels (SOC and PT).

To facilitate visualization and interpretation, temozolomide was chosen as the control drug. Given the relatively small number of reports for lomustine compared with bevacizumab and temozolomide, lomustine was only included in descriptive analyses and excluded from comparative analyses.

### 2.4 Adverse event coding

Adverse events were coded according to the System Organ Class (SOC) and Preferred Term (PT) categories based on the Medical Dictionary for Regulatory Activities (MedDRA) version 25.0. MedDRA provides a hierarchical structure consisting of five levels: Lowest Level Term (LLT), Preferred Term (PT), High Level Term (HLT), High Level Group Term (HLGT), and System Organ Class (SOC). In our study, adverse events associated with bevacizumab were identified at both the SOC and PT levels to characterize its safety profile. Subsequently, the safety profiles of bevacizumab and temozolomide were compared at both levels, using temozolomide as a reference. Specifically, an ROR value greater than 1 indicated a stronger association between the adverse event and bevacizumab relative to temozolomide, whereas an ROR value less than 1 indicated a weaker association.

## 3 Results

### 3.1 Descriptive analysis

**FIGURE 2:**
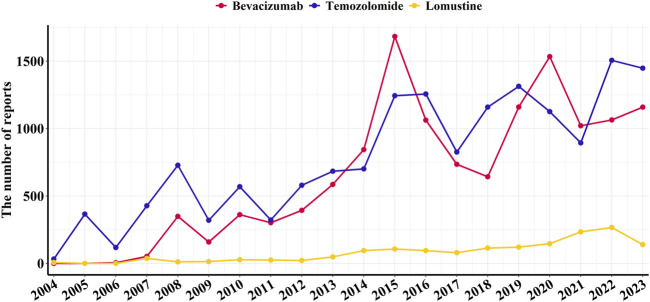
Annual number of reported adverse events associated with bevacizumab and alkylating agents from Q1 2004 to Q4 2023.

From Q1 2004 to Q4 2023, a total of 49,368,071 adverse event reports were extracted from the FAERS database, including 13,116 reports for bevacizumab, 15,621 reports for temozolomide, and 1,595 reports for lomustine (Figure 2). Data on adverse events related to the treatment of glioblastoma with these three drugs are shown in Figure 2.

It is noteworthy that bevacizumab has been used for glioblastoma treatment primarily within the past decade; therefore, adverse event reports for bevacizumab were not available for every year. The temporal distribution of adverse event reports peaked in 2015 and 2017, with 332 and 341 reports, respectively (Table 1).

**TABLE 1:** Summary of adverse event reports related to bevacizumab and alkylating agents from Q1 2004 to Q4 2023.

	Bevacizumab	Alkylating agent	
		Temozolomide	Lomustine
Number of events	3,323	5,283	427
Year			
2004	0	30	5
2005	0	145	0
2006	2	21	0
2007	24	84	7
2008	82	138	3
2009	74	99	6
2010	118	115	8
2011	65	78	1
2012	115	160	6
2013	169	185	10
2014	220	237	13
2015	332	389	28
2016	303	408	18
2017	341	385	26
2018	232	460	50
2019	233	426	42
2020	275	401	36
2021	192	395	58
2022	246	584	59
2023	300	543	51

Table 2 presents the demographic characteristics of glioblastoma patients reporting adverse events. A slight predominance of male patients was observed, accounting for 47.2%, 45.1%, and 46.4% of the reports for bevacizumab, temozolomide, and lomustine, respectively. Most patients were aged between 18 and 75 years. Physicians were the primary reporters for all three drugs, with the majority of reports originating from the United States, followed by Canada (Table 3).

**TABLE 2:** Demographic characteristics of patients associated with bevacizumab and alkylating agents from Q1 2004 to Q4 2023.

	Bevacizumab (Number of reports, %)		Alkylating agent (Number of reports, %)			
			Temozolomide		Lomustine	
Gender						
Female	1,048 (31.5%)		2,100 (39.8%)		147 (34.4%)	
Male	1,567 (47.2%)		2,381 (45.1%)		198 (46.4%)	
unkown	708 (21.3%)		802 (15.2%)		82 (19.2%)	
Age						
≤18	214		299		30	
18–75	1825		3,411		290	
≥75	112		256		7	
unknow	1,172		-		-	
Reporter						
	Physician	1,382 (41.6%)	Physician	1863 (35.3%)	Physician	186 (43.6%)
	Other health-professional	718 (21.6%)	Other health-professional	1,357 (25.7%)	Other health-professional	92 (21.5%)
	Consumer	490 (14.7%)	Health professional	1,039 (19.7%)	Health professional	73 (17.1%)
	Health professional	562 (16.9%)	Consumer	638 (12.1%)	Consumer	36 (8.4%)
	Pharmacist	137 (4.1%)	Pharmacist	196 (3.7%)	Pharmacist	30 (7.0%)
	unknow	33 (1.0%)	unknow	190 (3.6%)	unknow	10 (2.3%)
Outcomes						
	Other serious/important medical event	1,122	Other serious/important medical event	1830	Other serious/important medical event	186
	Death	896	Hospitalization	1,364	Hospitalization	102
	Hospitalization	809	Death	1,059	Death	85
	Life-threatening		Life-threatening	294	Life-threatening	15
	Disability	28	Disability	29	Disability	3

**TABLE 3:** Global distribution of adverse event reports for bevacizumab and alkylating agents.

Reporter country	Bevacizumab (Number of reports)	Alkylating agent (Number of reports)	
		Temozolomide	Lomustine
United States	1,560	2,207	100
Canada	428	371	94
Japan	269	419	-
France	238	425	95
Spain	76	95	7
Great Britain	66	244	18
Germany	63	272	25
China	58	88	-
Italy	55	212	26
Australia	52	83	4

The adverse event profiles of the three drugs, summarized in Table 4, exhibited distinct patterns. The most frequently reported adverse events for bevacizumab were fatigue (n = 276), hypertension (n = 220), and headache (n = 199). For temozolomide, thrombocytopenia (n = 581), disease progression (n = 475), and drug ineffectiveness (n = 356) were most commonly reported. In the case of lomustine, the most frequently reported events were thrombocytopenia (n = 67), progression of malignant neoplasm (n = 38), and fatigue (n = 36).

**TABLE 4:** Top ten adverse events reported for bevacizumab and alkylating agents.

		Alkylating agent			
Bevacizumab	Number of reports	Temozolomide	Number of reports	Lomustine	Number of reports
Off Label Use	319	Thrombocytopenia	581	Thrombocytopenia	67
Fatigue	276	Disease Progression	475	Malignant Neoplasm Progression	38
Hypertension	220	Drug Ineffective	356	Fatigue	36
Headache	199	Neutropenia	296	Neutropenia	36
Seizure	161	Nausea	267	Off Label Use	29
Platelet Count Decreased	142	Malignant Neoplasm Progression	256	Product Use In Unapproved Indication	28
Diarrhoea	137	Fatigue	250	Drug Ineffective	27
Proteinuria	107	Product Use In Unapproved Indication	238	Disease Progression	25
Deep Vein Thrombosis	103	Off Label Use	167	Seizure	24
Weight Decreased	91	Vomiting	166	Eastern Cooperative Oncology Group Performance Status Worsened	24

Table 5 details the distribution of adverse event onset times. Both bevacizumab and lomustine were associated with adverse events occurring predominantly within 2 months of treatment initiation. The median time to onset was 56 days (IQR: 21–138) for bevacizumab and 54 days (IQR: 18–175) for lomustine. Temozolomide, however, demonstrated a trend toward earlier onset, with a significant increase in adverse event reports occurring within 30 days after administration, and a median time to onset of 37 days (IQR: 19–92).

**TABLE 5:** Time-to-onset (TTO) analysis of adverse events associated with bevacizumab and alkylating agents.

	Bevacizumab	Alkylating agent	
		Temozolomide	LOMUSTINE
TTO (days)	56 (IQR: 21–138.75)	37 (IQR: 19–92)	54 (IQR: 18–175)
0–30 days (Number of reports)	332	708	36
31–60 days (Number of reports)	176	379	17
61–90 days (Number of reports)	109	163	11
≥90 (Number of reports)	349	428	37

### 3.2 Distribution of safety signals in glioblastoma patients treated with bevacizumab

Table 6 shows that a total of 174 safety signals (at the PT level) were identified across 22 SOCs for bevacizumab. The SOCs with the highest numbers of positive signals were “Investigations” (30 PTs, 17.24%), “Nervous system disorders” (30 PTs, 17.24%), and “Gastrointestinal disorders” (16 PTs, 9.19%). Notably, no safety signals related to neutropenia, febrile neutropenia, or hematotoxicity were observed among all PTs associated with bevacizumab, compared to the two alkylating agents (Table 7).

**TABLE 6:** Identified safety signals in glioblastoma patients treated with bevacizumab.

System Organ category	Preferred term	Number	ROR	95%CI lower ROR
Investigations	Platelet count decreased	142	1.28	1.05
	Weight decreased	91	2.08	1.6
	Blood pressure increased	87	3.5	2.59
	Weight increased	74	2.65	1.95
	Body temperature decreased	35	10.59	5.38
	Red cell distribution width increased	31	7.94	4.15
	Eastern cooperative oncology group performance status worsened	30	2.12	1.34
	Blood pressure systolic increased	28	13.31	5.81
	Heart rate decreased	21	4.99	2.54
	Oxygen saturation decreased	21	1.75	1.03
	Blood pressure diastolic increased	21	11.64	4.7
	Mean cell haemoglobin increased	19	4.86	2.4
	Neutrophil count increased	17	2.26	1.22
	Mean cell volume increased	16	6.65	2.85
	Protein urine present	13	3.93	1.76
	Blood pressure diastolic abnormal	12	39.9	5.19
	Monocyte count increased	9	2.49	1.05
	Protein urine	9	5.98	2
	Bacterial test positive	8	2.95	1.14
	Mean cell haemoglobin concentration decreased	8	13.29	2.82
	Blood test abnormal	7	2.91	1.05
	Blood pressure abnormal	7	3.32	1.17
	Blood chloride increased	6	4.98	1.41
	Eosinophil count decreased	6	3.32	1.07
	Mean platelet volume decreased	6	9.97	2.01
	Haemoglobin	5	8.31	1.61
	Grip strength decreased	4	13.29	1.49
	Blood alkaline phosphatase decreased	4	6.65	1.22
	Weight abnormal	4	13.29	1.49
	Mean platelet volume increased	3	9.97	1.04
Nervous system disorders	Headache	199	1.56	1.31
	Seizure	161	1.7	1.41
	Cerebral haemorrhage	59	1.77	1.29
	Cerebrovascular accident	59	3.51	2.43
	Haemorrhage intracranial	41	1.47	1.01
	Balance disorder	38	1.66	1.13
	Hypoaesthesia	34	2.06	1.34
	Memory impairment	33	1.89	1.23
	Cerebral infarction	32	1.83	1.19
	Ischaemic stroke	18	3.99	2.01
	Incoherent	17	4.04	1.99
	Hypersomnia	15	3.56	1.72
	Subarachnoid haemorrhage	14	2.74	1.35
	Cerebral ischaemia	13	2.27	1.12
	Posterior reversible encephalopathy syndrome	12	3.32	1.49
	Central nervous system haemorrhage	11	12.19	3.4
	Brain injury	9	4.27	1.59
	Migraine	8	3.8	1.38
	Optic neuritis	7	3.88	1.3
	Pyramidal tract syndrome	7	23.26	2.86
	Haemorrhagic stroke	7	4.65	1.48
	Cerebral artery stenosis	6	19.94	2.4
	Neuralgia	6	3.32	1.07
	Intracranial aneurysm	5	5.54	1.32
	Peroneal nerve palsy	5	5.54	1.32
	Taste disorder	4	6.65	1.22
	Slow speech	4	6.65	1.22
	Intracranial haematoma	3	9.97	1.04
	Reflexes abnormal	3	9.97	1.04
	Quadriplegia	3	9.97	1.04
Gastrointestinal disorders	Diarrhoea	137	1.49	1.21
	Abdominal pain	74	1.56	1.18
	Gastrointestinal perforation	50	10.42	5.93
	Intestinal perforation	38	7.91	4.41
	Rectal haemorrhage	38	3.83	2.4
	Gastrointestinal haemorrhage	33	2.61	1.66
	Large intestine perforation	30	2.56	1.59
	Dyspepsia	22	2.03	1.19
	Tongue ulceration	15	8.31	3.22
	Gastrointestinal disorder	13	2.27	1.12
	Diverticular perforation	11	3.05	1.34
	Glossodynia	8	4.43	1.54
	Anal fistula	7	11.63	2.42
	Pneumoperitoneum	7	2.91	1.05
	Tongue geographic	5	5.54	1.32
	Hypoaesthesia oral	4	13.29	1.49
Musculoskeletal and connective tissue disorders	Muscular weakness	83	1.67	1.28
	Arthralgia	46	1.8	1.26
	Back pain	40	2.22	1.49
	Pain in extremity	39	2.13	1.42
	Mobility decreased	38	2.3	1.52
	Musculoskeletal pain	26	4.32	2.41
	Myalgia	22	1.74	1.04
	Osteonecrosis	21	3.04	1.68
	Muscle spasms	17	2.26	1.22
	Bone pain	13	4.8	2.05
	Arthritis	7	7.75	2
	Musculoskeletal chest pain	6	6.65	1.66
	Joint effusion	5	4.15	1.12
	Osteonecrosis of jaw	3	9.97	1.04
General disorders and administration site conditions	Fatigue	276	1.69	1.46
	Death	83	0.49	0.42
	Gait disturbance	64	1.52	1.13
	Pain	51	1.65	1.18
	Impaired healing	38	4.36	2.69
	Therapy non-responder	27	2.81	1.68
	Therapy partial responder	21	3.04	1.68
	Peripheral swelling	14	2.02	1.04
	Ulcer	8	8.86	2.35
	Therapeutic product effect decreased	6	9.97	2.01
	Feeling cold	6	6.65	1.66
	Injection site bruising	3	9.97	1.04
Infections and infestations	Nasopharyngitis	43	1.93	1.33
	Wound infection	30	1.58	1.02
	Diverticulitis	20	3.91	2.05
	Candida infection	16	4.84	2.24
	Strongyloidiasis	13	3.32	1.54
	Tooth abscess	10	4.15	1.64
	Peritonitis	8	3.8	1.38
	Epstein-barr virus infection	6	3.32	1.07
	Appendicitis perforated	5	8.31	1.61
	Erysipelas	5	4.15	1.12
	Vaginal infection	3	9.97	1.04
	Oesophageal infection	3	9.97	1.04
Injury, poisoning and procedural complications	Off label use	319	1.53	1.34
	Intentional product use issue	78	1.83	1.39
	Infusion related reaction	43	7.54	4.39
	Contusion	34	3.06	1.92
	Wound dehiscence	25	3.62	2.05
	Radiation necrosis	21	2.33	1.33
	Nerve injury	15	16.63	4.81
	Fracture	14	9.31	3.35
	Skin abrasion	11	12.19	3.4
	Product administration error	6	3.99	1.22
	Skin laceration	3	9.97	1.04
Vascular disorders	Hypertension	220	4.97	4.03
	Deep vein thrombosis	103	1.27	1.01
	Haemorrhage	62	2.35	1.69
	Thrombosis	47	2.3	1.59
	Embolism	44	2.36	1.6
	Embolism venous	20	9.5	4.02
	Poor venous access	6	4.98	1.41
	Ischaemia	6	9.97	2.01
Skin and subcutaneous tissue disorders	Skin ulcer	44	9.16	5.17
	Alopecia	27	1.6	1.01
	Skin striae	17	5.14	2.41
	Skin irritation	9	9.97	2.7
	Skin necrosis	9	3.32	1.32
	Skin atrophy	5	8.31	1.61
	Scab	5	8.31	1.61
Neoplasms benign, malignant and unspecified	Tumour haemorrhage	34	1.59	1.06
	Neoplasm	31	1.95	1.25
	Malignant glioma	30	3.33	2
	Tumour necrosis	25	4.38	2.41
	Metastases to central nervous system	9	2.49	1.05
	Gliomatosis cerebri	3	9.97	1.04
Psychiatric disorders	Depression	31	1.66	1.08
	Sleep disorder	12	3.07	1.4
	Frustration tolerance decreased	5	8.31	1.61
	Bruxism	4	6.65	1.22
	Drug dependence	4	13.29	1.49
	Fear	3	9.97	1.04
Renal and urinary disorders	Proteinuria	107	17.06	10.68
	Nephrotic syndrome	13	21.61	4.88
	Nephrolithiasis	9	2.49	1.05
	Urine abnormality	9	14.96	3.23
	Micturition urgency	4	13.29	1.49
	Chronic kidney disease	3	9.97	1.04
Blood and lymphatic system disorders	Myelosuppression	53	6.31	3.99
	Bone marrow disorder	16	7.6	3.13
	Thrombotic microangiopathy	7	5.82	1.7
	Bicytopenia	4	13.29	1.49
Respiratory, thoracic and mediastinal disorders	Epistaxis	70	2.62	1.92
	Dysphonia	44	5.86	3.59
	Rhinorrhoea	17	2.46	1.31
	Upper-airway cough syndrome	3	9.97	1.04
Metabolism and nutrition disorders	Decreased appetite	74	1.42	1.08
Hepatobiliary disorders	Hepatocellular injury	31	2.1	1.34
Eye disorders	Optic neuropathy	18	3.99	2.01
Cardiac disorders	Myocardial infarction	11	2.28	1.06
Metabolism and nutrition disorders	Hyperkalaemia	11	3.05	1.34
Immune system disorders	Haemophagocytic lymphohistiocytosis	7	3.88	1.3
Social circumstances	Immobile	6	9.97	2.01
Ear and labyrinth disorders	Ear discomfort	4	13.29	1.49

**TABLE 7:** Comparative analysis of safety signal distribution between bevacizumab and alkylating agents.

PTs	Temozolomide		Lomustine		Bevacizumab	
	Number	95%CI lower limitation	Number	95%CI lower limitation	Number	95%CI lower limitation
Thrombocytopenia	581	2.67	67	1.78	7	1.7
Neutropenia	296	2.49	36	1.7	/	/
Pancytopenia	153	1.37	13	0.68	/	/
Lymphopenia	141	2.49	4	0.2	/	/
Leukopenia	118	1.25	11	0.68	/	/
Bone Marrow Failure	113	1.64	7	0.46	/	/
Febrile Neutropenia	109	1.55	20	1.88	/	/
Anaemia	98	0.92	5	0.23	/	/
Haematotoxicity	67	2.59	7	1.05	/	/
Myelosuppression	62	5.15	4	0.66	53	3.99
Agranulocytosis	48	3.75	4	0.52	/	/
Aplastic Anaemia	42	1.26	/	/	/	/
Febrile Bone Marrow Aplasia	18	0.75	/	/	/	/
Bone Marrow Disorder	18	3.52	/	/	16	3.13
Disseminated Intravascular Coagulation	14	0.52	/	/	/	/
Blood Disorder	9	0.69	/	/	/	/
Eosinophilia	9	0.83	/	/	/	/
Haemolytic Anaemia	7	0.56	/	/	/	/
Leukocytosis	5	0.76	/	/	/	/
Cytopenia	5	0.88	/	/	/	/
White Blood Cell Disorder	4	0.56	/	/	/	/
Aplasia Pure Red Cell	4	0.96	/	/	/	/
Thrombotic Microangiopathy	3	0.26	/	/	/	/
Bicytopenia	3	0.66	/	/	4	1.49
Platelet Disorder	3	0.38	/	/	/	/
Lymphadenopathy	3	0.22	/	/	/	/

### 3.3 Comparative safety analysis of bevacizumab versus temozolomide at the SOC level

Bevacizumab was compared with temozolomide at the SOC level, using temozolomide as the reference drug. Several SOCs demonstrated positive signals in glioblastoma patients treated with bevacizumab, including “Musculoskeletal and connective tissue disorders” (ROR = 2.77, 95% CI: 2.46–3.12), “Vascular disorders” (ROR = 2.17, 95% CI: 1.98–2.39), “Renal and urinary disorders” (ROR = 1.63, 95% CI: 1.43–1.87), “Nervous system disorders” (ROR = 1.46, 95% CI: 1.38–1.53), and “Investigations” (ROR = 1.36, 95% CI: 1.29–1.43).

**FIGURE 3:**
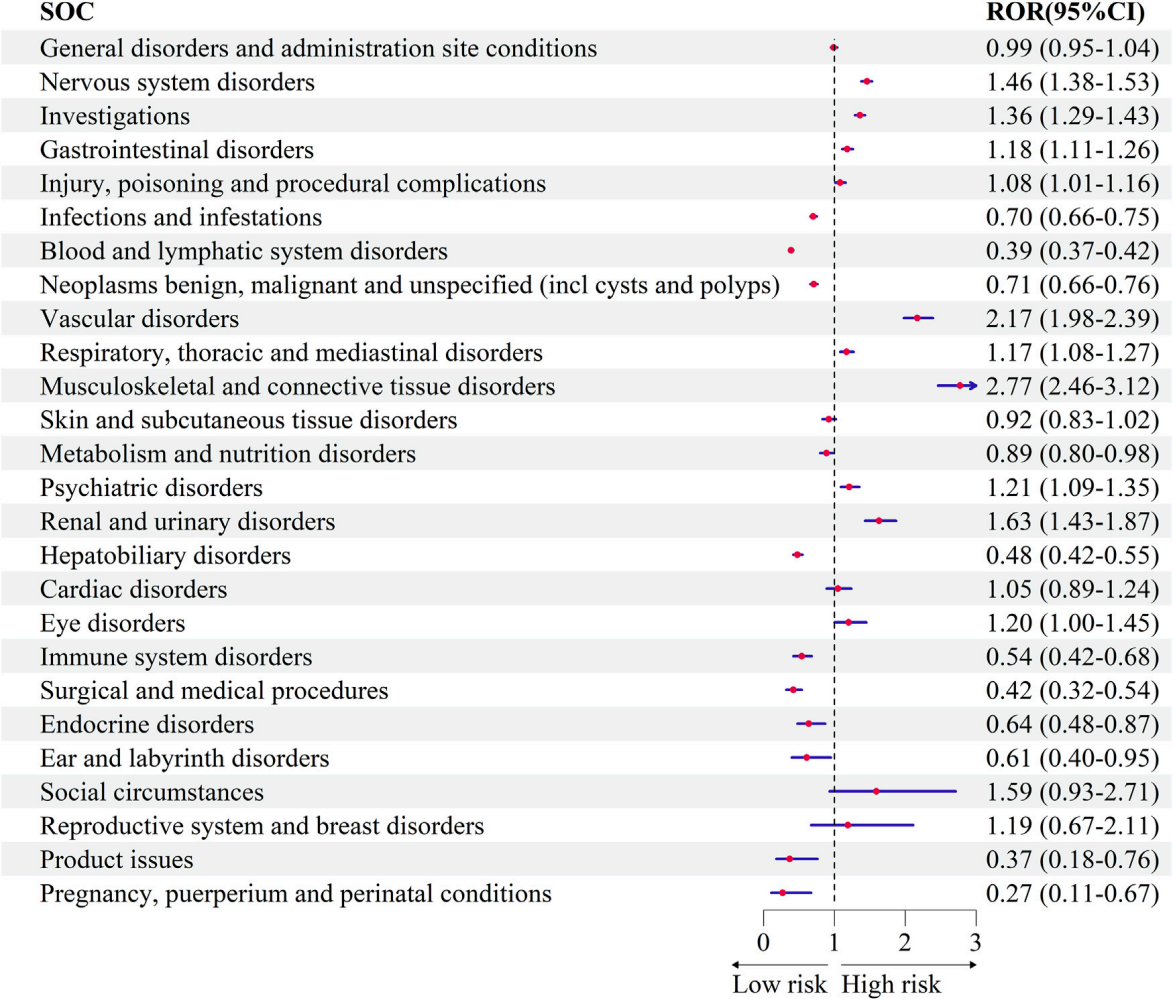
Comparative safety profiles across System Organ Classes (SOCs) for bevacizumab versus temozolomide.

Conversely, bevacizumab was associated with lower odds of adverse events in SOCs such as “Pregnancy, puerperium and perinatal conditions” (ROR = 0.27, 95% CI: 0.11–0.67), “Product issues” (ROR = 0.37, 95% CI: 0.18–0.76), “Surgical and medical procedures” (ROR = 0.42, 95% CI: 0.32–0.54), “Blood and lymphatic system disorders” (ROR = 0.39, 95% CI: 0.37–0.42), and “Ear and labyrinth disorders” (ROR = 0.61, 95% CI: 0.39–0.95) (Figure 3).

### 3.4 Comparison of safety signals at the PT level between bevacizumab and temozolomide

**FIGURE 4:**
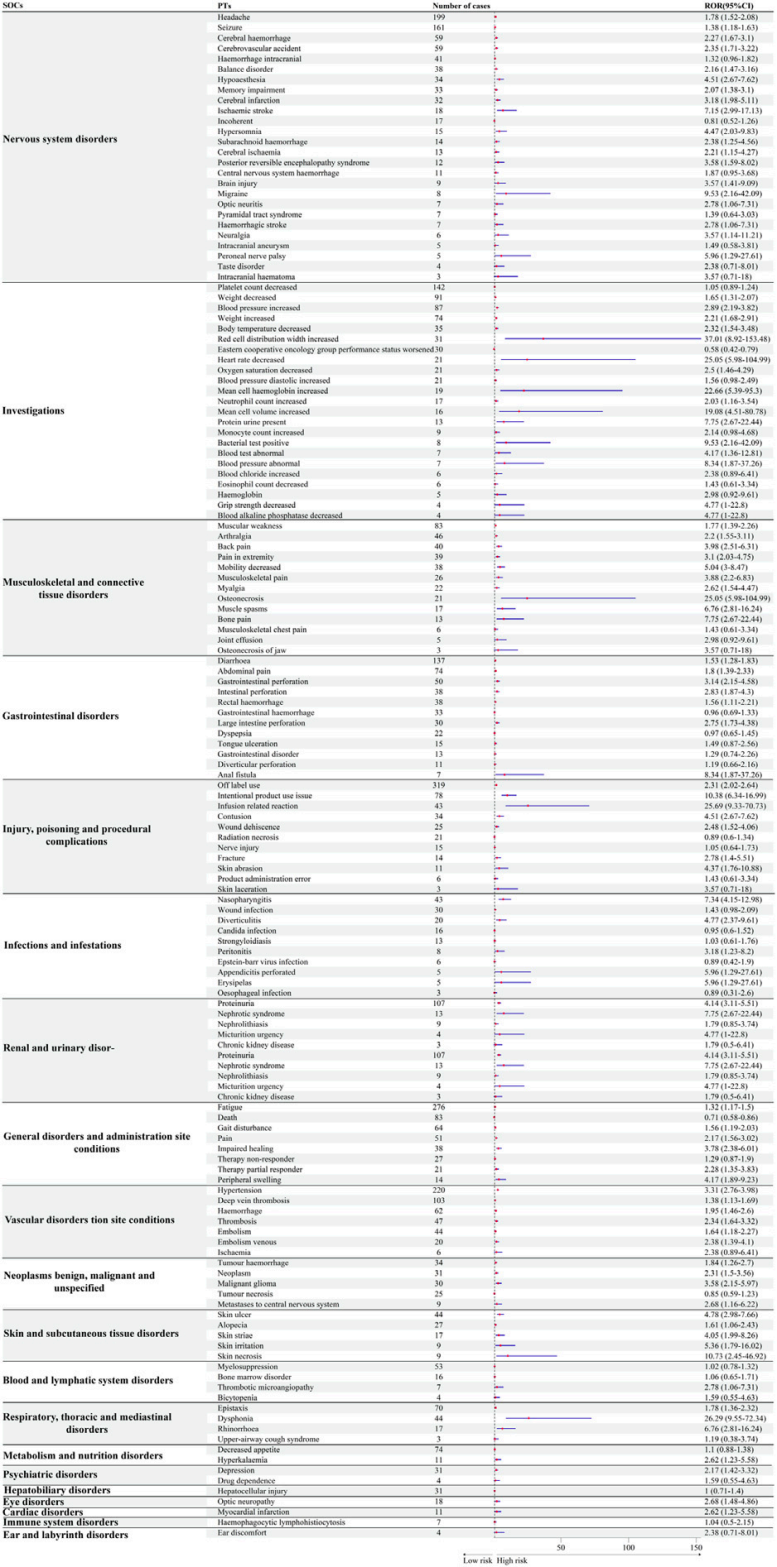
Comparative safety profiles at the Preferred Term (PT) level: safety signals of bevacizumab compared with temozolomide.

To further explore differences in safety signals between bevacizumab and temozolomide, we analyzed signal intensities at the PT level. Figure 4 shows that bevacizumab was strongly associated with certain adverse events compared to temozolomide, including dysphonia (Respiratory, thoracic and mediastinal disorders) (ROR = 26.28, 95% CI: 9.55–72.34), perfusion-related reactions (Injury, poisoning and procedural complications) (ROR = 25.68, 95% CI: 9.33–70.23), and increased erythrocyte distribution width (Investigations) (ROR = 37.01, 95% CI: 8.92–153.48).

## 4 Discussion

Glioblastoma is the most common and aggressive primary malignant brain tumour (Stupp et al., 2007). Temozolomide and lomustine are DNA-alkylating agents, while bevacizumab is a humanised monoclonal antibody against vascular endothelial growth factor that inhibits angiogenesis. Both alkylating agents and bevacizumab are currently first-line treatments for glioblastoma (Pombo Antunes et al., 2020). The increasing number of adverse event reports associated with alkylating agents and bevacizumab between 2004 and 2023 highlights the complexity of glioblastoma treatment. By 2023, the annual number of reports had nearly tripled, underscoring the critical need for rigorous post-marketing surveillance to safeguard patient health.

In this study, temozolomide was reported significantly more frequently than bevacizumab, suggesting wider use in glioblastoma treatment (Lee, 2016). Bevacizumab was approved by the U.S. Food and Drug Administration (FDA) in 2009 for glioblastoma and is also included in the European Association of Neuro-Oncology (EANO) guidelines (Fu et al., 2023). Reports of adverse events related to bevacizumab peaked in 2015 and 2017, with 332 and 341 cases, respectively, possibly reflecting its increasing global uptake after approval. The United States led in the number of bevacizumab-related reports, potentially due to earlier market approval, broader expansion of indications, and greater patient engagement in reporting adverse events. In contrast, bevacizumab was approved several years later in other countries (Funakoshi et al., 2020; Australian Government Department of Health and Aged Care, 2019).

Our findings are consistent with, and extend, previous clinical trials on bevacizumab. Specifically, the highest number of significant safety signals for bevacizumab were observed in the “nervous system disorders” category (30 events, 17.24%). The NRG Oncology RTOG 0825 study by Wefel et al. showed that adding bevacizumab to standard chemotherapy did not improve survival endpoints but did significantly worsen neurocognitive function over time (Wefel et al., 2021). Similarly, Rodríguez et al. reported lower neurocognitive test scores among glioblastoma patients treated with bevacizumab compared to placebo (Rodriguez-Camacho et al., 2022). Hypertension, proteinuria, bone marrow failure, and leukopenia are among the most common adverse events listed on the bevacizumab label. In our study, adverse events such as elevated blood pressure, increased systolic and diastolic pressure were notably prominent, in line with previous reports estimating hypertension affects about 40% of patients treated with bevacizumab (Carvalho et al., 2020; Khan et al., 2022). A randomized controlled trial also reported hypertension and headache in over 10% of glioblastoma patients receiving bevacizumab (Cloughesy et al., 2020). Both the EMA and FDA have identified hypertension, fatigue, and malaise as the most frequent adverse events (European Medicines Agency, 2017; United States Food and Drug Administration (U.S. FDA)), underscoring the importance of continuous blood pressure monitoring during treatment. Additionally, significant positive signals were detected for thrombotic events such as deep vein thrombosis, thrombosis, and thrombotic microangiopathy, aligning with earlier studies observing vascular adverse events associated with bevacizumab (Oki et al., 2018; Perry, 2012). Ranpura et al. further reported an increased risk of venous thromboembolism in bevacizumab-treated patients (Ranpura et al., 2011). Interestingly, we also identified safety signals not mentioned in the official prescribing information, including psychiatric-related adverse events such as depression, sleep disturbances, and decreased frustration tolerance. These findings suggest the need for healthcare providers to remain vigilant for emerging safety signals to optimize prescribing practices and improve patient management.

Our results indicate that glioblastoma patients treated with bevacizumab may have a higher risk of adverse events in specific System Organ Classes (SOCs) compared with those treated with temozolomide, particularly “musculoskeletal and connective tissue disorders” (ROR = 2.77, 95% CI: 2.46–3.12), “vascular disorders” (ROR = 2.17, 95% CI: 1.98–2.39), and “renal and urinary disorders” (ROR = 1.63, 95% CI: 1.43–1.87). These findings do not suggest that bevacizumab should be avoided, but rather that further research is warranted to better understand these risks. Chinot et al. reported that adding bevacizumab to radiotherapy and temozolomide did not improve survival in glioblastoma patients, while the incidence of adverse events was higher in the bevacizumab group, particularly arterial thromboembolic events, bleeding, wound healing complications, gastrointestinal perforation, and congestive heart failure (Chinot et al., 2014). Notably, bevacizumab demonstrated a lower signal for certain SOCs compared to temozolomide, including “pregnancy, puerperium and perinatal conditions” (ROR = 0.27, 95% CI: 0.11–0.67), “blood and lymphatic system disorders” (ROR = 0.39, 95% CI: 0.37–0.42), and “ear and labyrinth disorders” (ROR = 0.61, 95% CI: 0.39–0.95). Moreover, bevacizumab was not associated with positive safety signals for neutropenia, febrile neutropenia, or hematological toxicity, suggesting a potentially lower risk of hematological disorders compared to temozolomide. If confirmed in future studies, these findings could represent a meaningful therapeutic advantage for glioblastoma patients.

Given the urgent need for novel therapeutic approaches, promising new avenues are being explored. SurVaxM, a survivin-targeted peptide vaccine, has demonstrated good tolerability in combination with temozolomide, with the most common adverse event being injection site inflammation, which was self-limited (Ahluwalia et al., 2023). Similarly, the CheckMate 498 study reported fatigue as the most common adverse event during treatment with nivolumab and radiotherapy, with a neurological adverse event rate of 16.5% (Omuro et al., 2023). These advances highlight the importance of keeping abreast of emerging therapies in glioblastoma management.

In our study, male patients accounted for 47.2%, 45.1%, and 46.4% of adverse event reports related to bevacizumab, temozolomide, and lomustine, respectively. This aligns with epidemiological data showing glioblastoma incidence is 1.58 times higher in men (Weller and Le Rhun, 2020; Tan et al., 2020). Therefore, special attention should be given to male patients when prescribing these therapies.

We also observed that adverse events associated with bevacizumab occurred over a longer duration compared with temozolomide. Temozolomide was associated with a shorter time to adverse event onset, typically peaking around 30 days post-treatment initiation, consistent with previous reports (Villano et al., 2012). Meanwhile, adverse events related to bevacizumab generally emerged within 50 days of starting treatment (Gerriets and Kasi, 2025). These findings suggest a need for heightened vigilance early in temozolomide therapy.

Despite the insights provided, this study has several limitations. First, the FAERS database is a spontaneous reporting system susceptible to reporting bias, duplication, and inaccuracies, potentially skewing safety assessments. Second, FAERS data only reflect a snapshot in time, with ongoing changes in report numbers. Third, the lack of detailed comorbidity and treatment information, including precise medical histories, limits interpretability. Fourth, the actual incidence rates cannot be calculated due to the absence of denominator data (i.e., total number of drug users). Most importantly, FAERS does not provide severity ratings based on the Common Terminology Criteria for Adverse Events (CTCAE), restricting clinical severity comparisons. Nevertheless, our findings provide valuable guidance for future research and clinical practice.

The distinct safety profiles of alkylating agents and bevacizumab carry important clinical implications. For instance, bevacizumab’s lower incidence of hematological adverse events may make it a preferable choice for patients with pre-existing hematological conditions. Additionally, the delayed onset of bevacizumab-related adverse events may favor its use in patients with complex comorbidities who cannot tolerate early toxicities. The novelty of this study lies in its extensive use of the FAERS database to systematically compare the safety profiles of alkylating agents and bevacizumab. Our results emphasize the urgent need for continuous post-marketing surveillance and evaluation. Future research should incorporate prospective designs and integrate genetic and biomarker data to personalize glioblastoma therapy and elucidate the mechanisms behind observed adverse events.

Over the 20-year period from 2004 to 2023, bevacizumab demonstrated fewer overall adverse events than temozolomide for glioblastoma treatment. While bevacizumab may have certain safety advantages, particularly regarding hematological disorders, vigilance regarding hypertension and vascular events remains essential. Ultimately, our findings aim to support therapeutic decision-making, although further follow-up studies, treatment interruption analyses, and pharmacokinetic evaluations are required to confirm causality.

## Data Availability

The original contributions presented in the study are included in the article/supplementary material, further inquiries can be directed to the corresponding author.

## Abbreviations

CI, Confidence Interval; EMA, European Medicines Agency; FAERS, FDA Adverse Event Reporting System; HLT, High level term; HLGT, High group level term; LLT, Lowest level term; MedDRA, Medical Dictionary for Regulatory Activities; PT, Preferred Terminology; ROR, Ratio of reported ratios; SOC, System Organ Class; VEGF, Vascular epithelial growth factor.
